# Successful treatment with denosumab for pelvic fibrous dysplasia: A case report and review of the literature

**DOI:** 10.1097/MD.0000000000028138

**Published:** 2021-12-10

**Authors:** Kunihiro Ikuta, Tomohisa Sakai, Hiroshi Koike, Kan Ito, Shiro Imagama, Yoshihiro Nishida

**Affiliations:** a Department of Orthopedic Surgery, Nagoya University Graduate School and School of Medicine, 65 Tsurumai, Showa, Nagoya, Japan; b Department of Rehabilitation, Nagoya University Hospital, 65 Tsurumai, Showa, Nagoya, Japan.

**Keywords:** cystic fibrous dysplasia, denosumab, pelvis, tartrate-resistant acid phosphatase 5b

## Abstract

**Rationale:**

Fibrous dysplasia is a rare disorder that results in fractures, pain, and disability and can affect any bone in the body. The treatment of symptomatic fibrous dysplasia is determined based on the affected bones. Although some lesions are often too extensive for surgical procedures, there are currently no effective or recommended medical treatments available for them.

**Patient concerns:**

A 27-year-old woman developed right buttock pain and was diagnosed with a bone tumor in the right ilium. Clinical images revealed an expansive osteolytic lesion with thinning of the cortex and cystic change from the acetabulum to the sacroiliac joint.

**Diagnosis:**

An incisional biopsy was performed, and the lesion was diagnosed as cystic fibrous dysplasia. Occasional osteoclast-like giant cells and woven bone were observed. The patient had no evidence of polyostotic lesions or findings of McCune-Albright syndrome. Biochemical blood test results showed no obvious abnormal values, except for an increase in serum tartrate-resistant acid phosphatase 5b to 459 mU/dL.

**Interventions:**

Since surgical treatment appeared to be challenging, she was treated with denosumab with decreased dose-intensity schedules.

**Outcomes:**

The administration of denosumab caused osteosclerosis within the lesion, resulting in the elimination of bone pain. The patient received denosumab treatment for 18 months. Pain relief and lesion radiodensity were maintained for 9 months after denosumab discontinuation. The serum level of tartrate-resistant acid phosphatase 5b was measured to monitor the response to denosumab, which was suppressed during denosumab treatment.

**Lessons:**

We described successful denosumab treatment in a patient with cystic fibrous dysplasia (FD) who maintained efficacy for 9 months after treatment. Although the use of denosumab in fibrous dysplasia is currently off-label, our experience with this patient supports the potential of denosumab therapy for patients for whom surgical treatment is challenging.

## 1 Introduction

Fibrous dysplasia (FD) is a skeletal disorder characterized by the replacement of the trabecular bone with abnormal fibrous and immature osseous tissue. FD can be either monostotic or polyostotic and affects any bone in the body, most commonly the femur, tibia, ribs, skull, humerus, and pelvis. The polyostotic type is often associated with McCune-Albright syndrome (MAS), which has café-au-lait macules and endocrinopathies or precocious puberty. FD involves a somatic mutation in *GNAS*, a gene encoding the Gsα protein, a guanine nucleotide-binding protein (G protein) that mediates intracellular signaling.^[1,2]^

Although most patients with FD experience few or no symptoms, surgical procedures are required for some patients with persistent pain, risk of pathological fracture, bone deformity, or prolonged fracture healing. These patients may undergo curettage and bone grafting, but the grafted bone tends to be resorbed in younger patients or those with the polyostotic type. Pain is more likely to occur if the lesion is located in a weight-bearing bone with thinning of the cortex. Significantly, the surgical treatment of pelvic lesions is challenging due to their anatomical complexity, difficulty in controlling hemorrhage, and risk of damage to the acetabulum or sacroiliac joint, which can impair pelvic or hip function. Therefore, alternative treatments are required for patients with symptomatic pelvic lesions.

Previous studies have implicated increased osteoclast activity in the pathophysiology of FD.^[3,4]^ Several reports have proposed that antiresorptive treatments, mainly intravenous bisphosphonates, would be helpful for decreasing bone pain in symptomatic FD.^[1,5]^ However, there is no evidence that bisphosphonates directly affect FD lesions by reducing their growth or disease activity.^[1,5]^ There are no guidelines for the optimal treatment of symptomatic FD when the lesion is unsuitable for surgical treatment.

Denosumab is a humanized monoclonal antibody that binds to the cytokine receptor activator of nuclear factor-kappa B ligand and prevents it from activating the RANK receptor of osteoclasts, thereby inhibiting osteoclast function. Previous in vitro studies have shown that Gsα mutations upregulate Receptor activator of nuclear factor-kappa B ligand expression in FD.^[6]^ Thus, denosumab may be useful in the treatment of FD and deserves further investigation. Treatment with denosumab has been reported to demonstrate a successful response in a small number of patients with FD.^[7–12]^ Here, we report a case of a young woman with monostotic FD in the right ilium that was treated with periodic administration of denosumab.

## 2 Case presentation

**Figure 1:**
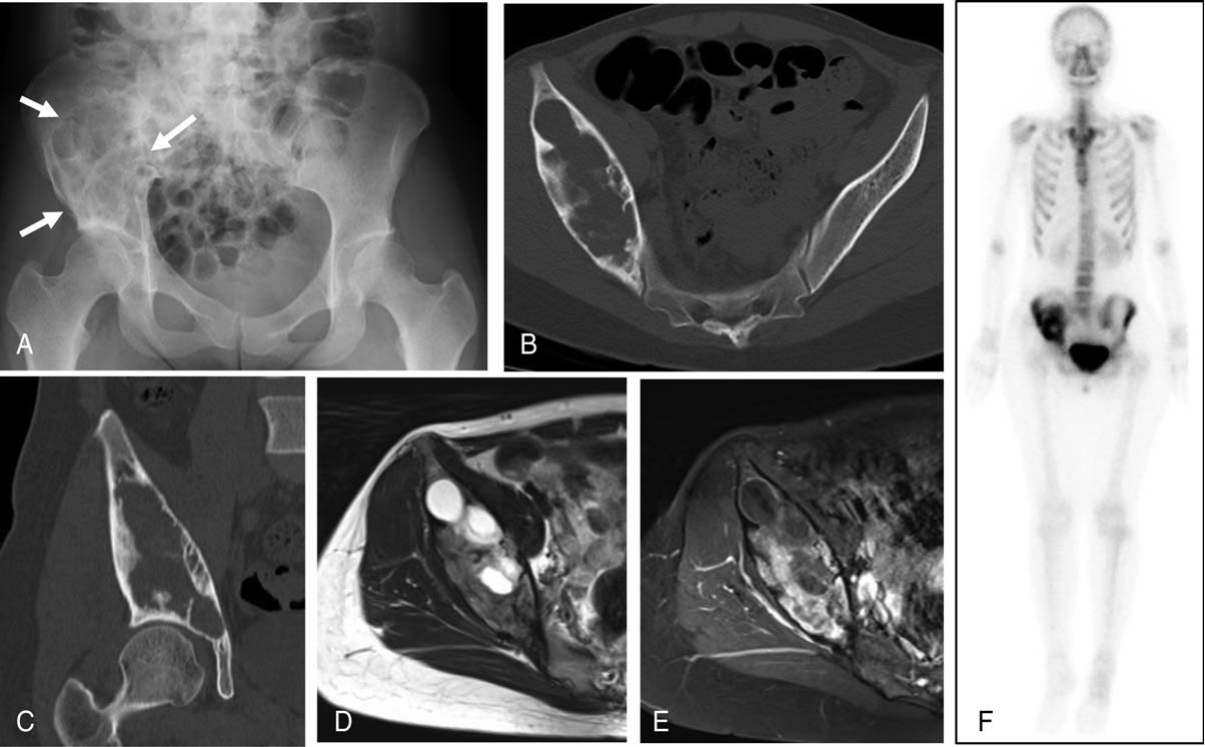
Images at initial visit (A) Plain radiograph showing a large osteolytic lesion (arrows) on the right ilium. (B) Axial view of pelvic computed tomography (CT) showing an expansile lytic lesion with cortical thinning. (C) Coronal CT showing the lesion extending to the subchondral bone of the acetabulum. (D) Axial T2-weighted magnetic resonance (MR) imaging revealed a multilocular lesion with high signal intensity, and (E) T1-fat suppressed axial MR image showing the lesion to be heterogeneously enhanced with gadolinium. (F) Bone scintigraphy showing increased focal uptake in the right ilium.

**Figure 2:**
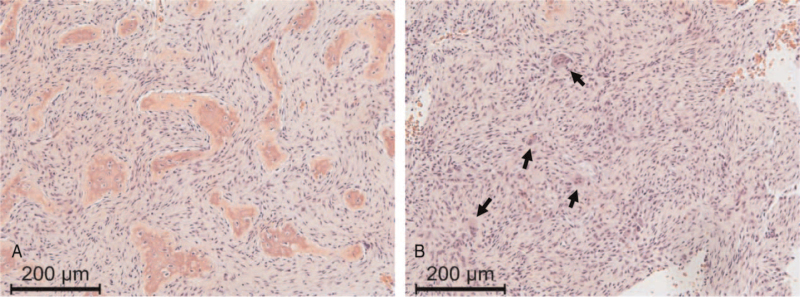
Histological examination of a biopsy specimen. (A) The proliferation of fibroblasts lacking atypical features and irregular trabeculae without osteoblastic rimming were observed. (B) Occasional osteoclast-like giant cells (arrows) and woven bone were present. Hematoxylin and eosin, ×100.

A 27-year-old woman was referred to our department with radiographs taken at a local clinic for persistent pain in her right hip after a skiing fall. At the time of her first visit, she was limping, and the range of motion of her right hip was limited. Plain radiography revealed a large osteolytic lesion in the right ilium (Fig. 1A). Computed tomography (CT) revealed an expanding lesion with thinning of the cortex from the acetabulum to the sacroiliac joint (Fig. 1b and 1c). Magnetic resonance imaging revealed no extra-skeletal masses. The lesion included areas showing an iso-signal intensity compared with that of skeletal muscle on T1-weighted images, a higher signal intensity on T2-weighted images, and an enhanced margin of the lesion with gadolinium, suggesting the presence of cystic degeneration (Fig. 1d and 1e). Clinical images were suggestive of bone tumors such as giant cell tumor of bone (GCTB), aneurysmal bone cyst, and FD. Histological examination following incisional biopsy showed irregular trabeculae with fibrous stroma (Fig. 2A) mixed with fibrous membrane tissues. Features of atypia were not found, while osteoclast-like giant cells were occasionally observed. (Fig. 2B). Based on these findings, a diagnosis of FD with cystic changes was made. Bone scintigraphy confirmed the presence of increased uptake in the right ilium (Fig. 1F). The patient had no evidence of polyostotic lesions or MAS symptoms. Her biochemical blood test results showed no obvious abnormal values, except for an increase in serum tartrate-resistant acid phosphatase 5b (TRACP-5b) to 459 mU/dL.

She could not work due to right hip pain, expressed as 8/10 on a numerical rating scale (NRS) at that time. Considering the invasiveness or the possibility of massive hemorrhage induced by surgical intervention, the lesion was not considered amenable to intralesional curettage and bone grafting. Considering the patient's preference, we decided on conservative therapy, and denosumab was administered according to the established protocol in GCTB under off-label use approval at our institution. The patient provided informed consent for the treatment with denosumab. Regular dental evaluations were also conducted.

**Figure 3:**
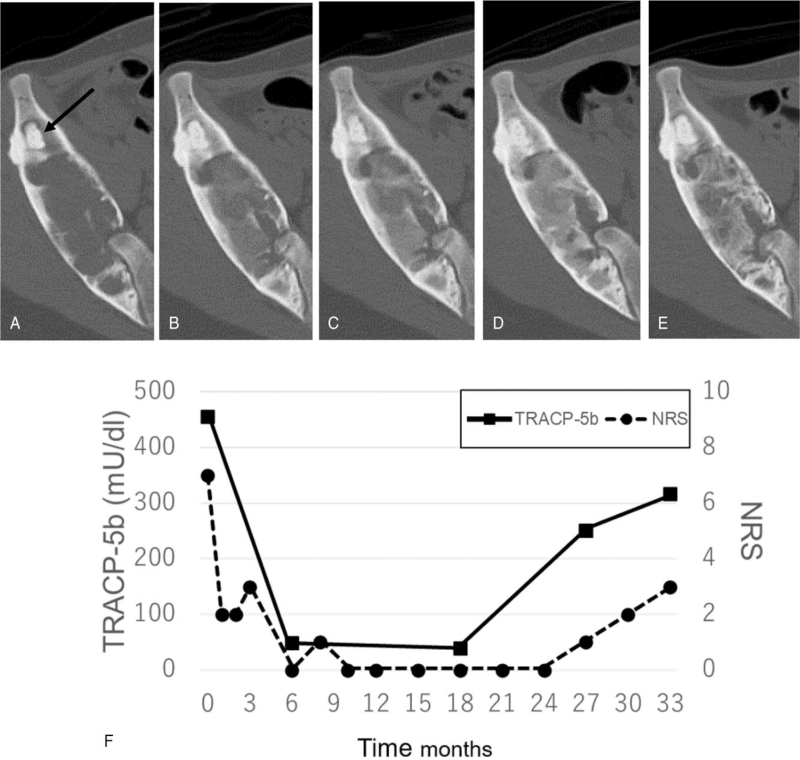
Radiographic evaluation with axial views of pelvic CT, and changes in pain scores and the serum level of TRACP-5b during and after denosumab treatment. (A) An expansive osteolytic lesion was observed at the start of denosumab administration. The presence of bone cement that had been used at the biopsy before administration of denosumab was noted (arrow). (B) The radiodensity within the lesion was slightly increased 3 months after denosumab administration. (C) The appearance of the lesion was more radiodense 6 months after denosumab administration. (D) Osteosclerotic changes in the lesion were observed 12 months after denosumab administration. (E) The radiolucent areas were increased within the lesion nine months after denosumab discontinuation (27 months after denosumab administration). (F) The serum level of TRACP-5b was correlated with pain scores as expressed with a numeric rating score (NRS).

Denosumab was administered at 120 mg/day on days 1, 8, 15, and 29, and monthly thereafter, in addition to daily calcium supplementation. After 4 weeks (day 29) of denosumab administration, the hip pain showed a marked decline (NRS 2/10), and she was able to return to work. Since hypocalcemia (7.9 mg/dL) was observed as a side effect of denosumab administration, vitamin D supplementation was started. CT images after 3 months of denosumab therapy revealed increased osteosclerotic areas within the osteolytic lesion (Fig. 3a and 3b). No reduction in the size of the affected areas was observed. After 3 months of treatment, denosumab administration was decreased to once every 2 months. Six months after denosumab treatment, the osteosclerotic changes in the lesion became more prominent (Fig. 3C), and the hip pain completely disappeared (NRS 0/10) (Fig. 3F). The serum TRACP-5b level decreased to below the lower limit of normal (49 mU/dL) after 6 months of denosumab treatment. Since the symptoms and disease status were stable, denosumab administration was decreased to once every 3 months from 12 months after the treatment (Fig. 3D). At 18 months of treatment, denosumab was discontinued because the patient wanted to become pregnant. The TRACP-5b level at that time was 40 mU/dL.

Biochemical blood tests were performed routinely after discontinuation of denosumab treatment, but hypercalcemia was not observed. CT revealed increased osteolytic changes within the lesion, and the hip pain flared up nine months after discontinuation of treatment (Fig. 3E). The TRACP-5b level remained within the normal range but increased to 251 mU/dL. The patient did not resume denosumab treatment because of her desire to have a baby. At the last visit, 15 months after denosumab discontinuation, she was able to work with mild hip pain (NRS 3/10) controlled without analgesics. The serum TRACP-5b level further increased to 318 mU/dL but was still below the upper limit of the normal range (Fig. 3F).

## 3 Discussion

Although polyostotic FD is commonly associated with flat bones, monostotic FD in the pelvis is rare.^[13]^ The typical radiographic finding of FD is a hazy, “ground glass-like” appearance. However, the presence of hemorrhage, secondary aneurysmal bone cyst formation, mucinous degeneration, and cystic changes can lead to diagnostic confusion. In our case, the radiographic features of the lesion depicted osteolysis and expansion with cystic changes and cortical thinning, which led us to consider the possibility of giant cell tumor of bone. Pathological examination revealed cystic FD. Cystic FD is not uncommon in the skull, facial bones, and ribs, and it has been sporadically reported in long tubular bones.^[14,15]^ Longstanding FD lesions can be accompanied by cystic changes.^[16]^ However, there have been no reports on how often FD lesions in the pelvis are associated with cystic changes.

In this case, the dosing intervals for denosumab were altered depending on the disease activity. Data on long-term denosumab administration were not obtained due to the patient's desire to have a baby, but efficacy may be observed even at longer dosing intervals. Several recent reports have shown that patients with refractory FD benefit from denosumab treatment with different schedules and doses (Table 1).^[7–12,17,18]^ Majoor et al suggested that a treatment regimen of 60 mg every 6 months was insufficient to achieve a sustained reduction of bone turnover markers in patients with FD, and all patients were eventually treated with 60 mg of denosumab every 3 months.^[9]^ Favorable clinical responses to treatment with denosumab in these reports and our case support further investigation regarding the potential use of denosumab in patients with refractory FD. On the other hand, it was recommended that denosumab be used in specialized facilities, preferably within the framework of clinical studies or trials in the guidelines from the International FD/MAS International Consortium.^[1]^ A clinical trial in FD is currently underway to evaluate the clinical efficacy and histological effects of denosumab treatment, including the biochemical response after discontinuation (NCT03571191). In the trial, the dosage and dosing intervals followed the protocol of GCTB administered to our patient in the first 3 months of treatment.

**Table 1:** Cases with FD treated with denosumab in the previous literature.

References	Number of patients	FD type	Treatment	Effect on bone pain	Bone resorption marker	Radiographic improvement	BPs prior to denosumab treatment	Adverse events	Duration of denosumab treatment
Boyce, 2012^[12]^	1	MAS	1 mg/kg, every 3 M	Yes	CTX	No	Yes	Hypercalcemia after discontinuation	7 M
Ganda, 2013^[10]^	2	PFD (2)	60 mg, every 6–9 M (case1), every 4 M (case2)	Yes	uDPD	NA	Yes	None	NA
Eller-Vainicher, 2016^[7]^	1	MFD	60 mg, every 3 M	Yes	CTX	Yes	Yes	None	27 M
Majoor, 2019^[9]^	12	PFD (7) MAS (4) MFD (1)	60 mg, every 3–6 M	Yes (10)	CTX	NA	Yes	Skin rash (1)	median 15.5 M
Meier, 2021^[17]^	37	PFD (21) MAS (9) MFD (7)	60 mg, every 3–6 M	Yes^∗^	CTX	NA	Yes (34)	Osteonecrosis of jaw (1), oral blisters (1)	median 1.6 yr
van der Bruggen, 2021^[18]^	8	PFD (5) MAS (2) MFD (1)	60–120 mg, every 3–6M	NA	NA	Yes	No	NA	NA
Raborn, 2021^[11]^	1	MFD	53–70 mg, every 1–3M	Yes	CTX	Yes	Yes	Hypercalcemia after discontinuation	3.5 yr
Meier, 2021^[8]^	2	PFD (2)	60 mg, every 3M	Yes	CTX	Yes	Yes	None	5.3–5.5 yr

In patients with unresectable or metastatic GCTB, control of the disease by prolonged administration is attempted in a clinical setting. However, even in GCTB, for which denosumab has been approved, there is still a lack of evidence regarding the optimal duration of treatment, appropriate dosing intervals, and risks of long-term administration for patients requiring long-term disease control. It may be acceptable to use denosumab only during pain reactivation to reduce the risk of adverse events. Hypercalcemia after denosumab discontinuation has been reported in younger patients with GCTB. Regarding FD, Boyce et al. and Raborn et al reported an increased risk of significant hypercalcemia after treatment cessation in children.^[11,12]^ Meier et al. investigated adverse events of denosumab (60 mg every 3–6 months) in 37 patients with FD and found that 1 patient (2.7%) had hypercalcemia 5 months after its discontinuation.^[17]^ In the present case, close clinical monitoring with laboratory data was performed after discontinuation of denosumab treatment. No increase in serum calcium level was observed. In a study by Meier et al, 1 patient (2.7%) with FD developed osteonecrosis of the jaw with a median cumulative dose of 660 mg.^[17]^ Palmerini et al reported a 6% incidence of osteonecrosis of jaw in 97 patients with GCTB who were treated with denosumab for a median of 20 months.^[19]^

TRACP-5b has been used as a marker of bone resorption in patients with osteoporosis or GCTB. The normal value of TRACP-5b ranges from 120 to 420 mU/dL in females. In this case, denosumab treatment with decreased dose-intensity schedules significantly suppressed TRACP-5b levels and increased osteosclerosis within the lesion, which resulted in the elimination of bone pain. However, the patient had progression of disease nine months after cessation of denosumab treatment. The osteolytic areas in the CT images were enlarged again, the pain recurred, and the serum TRACP-5b level was still within normal limits but increased to 251 mU/dL. At the last follow-up, 15 months after denosumab discontinuation, persistent pain and progressive osteolytic change coincided with a further increase in TRACP-5b to 318 mU/dl. Serial measurements of TRACP-5b showed that its serum level reflected the disease activity of cystic FD. This finding supports the contention that TRACP-5b can serve as a surrogate treatment marker to help determine the dose and duration of denosumab therapy in patients with FD.

## 4 Conclusion

We described successful denosumab treatment in a patient with cystic FD who maintained efficacy for nine months after treatment. In addition, the finding of increased osteosclerotic lesions supports the contention that denosumab may directly affect FD lesions. Thus, TRACP-5b may be a useful indicator for evaluation the disease activity of FD. Although the use of denosumab in FD is currently off-label, the information in this report supports the consideration of denosumab in the treatment algorithm for patients with FD unsuitable for surgical treatment.

## Acknowledgment

The authors thank Satoko Shimada, a pathologist of Nagoya University Hospital, for the pathological diagnosis of the disease.

## Author contributions

**Conceptualization:** Kan Ito.

**Data curation:** Kunihiro Ikuta, Hiroshi Koike.

**Funding acquisition:** Kunihiro Ikuta.

**Methodology:** Yoshihiro Nishida.

**Supervision:** Shiro Imagama.

**Visualization:** Kunihiro Ikuta, Tomohisa Sakai.

**Writing – original draft:** Kunihiro Ikuta.

**Writing – review & editing:** Shiro Imagama, Yoshihiro Nishida.
